# Why do psychotherapists use so little e-mental health in psychotherapy? Insights from a sample of psychotherapists in Germany

**DOI:** 10.1371/journal.pmen.0000270

**Published:** 2025-03-19

**Authors:** Sarah Wüllner, Tobias Hecker, Pia Flottmann, Katharin Hermenau

**Affiliations:** 1 University Clinic of Child and Adolescent Psychiatry and Psychotherapy, Protestant Hospital Bethel, Bielefeld University, Medical School EWL, Bielefeld, Germany; 2 Institute for Interdisciplinary Conflict and Violence Research, Bielefeld University, Bielefeld, Germany; 3 Department of Psychology, Faculty of Psychology and Sports Science, Bielefeld University, Bielefeld, Germany; Instituto Federal do Maranhão: Instituto Federal de Educacao Ciencia e Tecnologia do Maranhão, BRAZIL

## Abstract

During the COVID-19 pandemic, psychotherapists had to use e-mental health to continue their treatment and to stay in contact with patients. Even after the pandemic, many psychotherapists continued to use modern technologies such as videoconferencing. However, the pandemic did not lead to increased use of all types of e-mental health. The aim of the present study was to assess the usage of and potential association factors with e-mental health in a sample of German psychotherapists. We focused on the use of videoconferencing and mental health apps for e-mental health use. This was an online survey study. The participants were 159 German psychotherapists with an average age of 44.02 years (SD=13.18). The survey consisted of questions about the primary psychotherapeutic approach, treating minors or adults, attitudes toward e-mental health and the individual use of modern technologies and e-mental health in private and professional contexts. In the current sample, the utilization of mental health apps is far from being integrated into daily routines, with 82% of the psychotherapists not recommending mental health apps in psychotherapy. The majority of the psychotherapists had limited technical equipment available at their workplace. The psychotherapeutic approach, potential to augment psychotherapy and technical equipment available at work were significant correlates of the therapeutic range of e-mental health use. To address low e-mental health use, it is necessary to understand the correlates of e-mental health use within different levels of use.

## 1. Introduction

The COVID-19 pandemic was a catalyst for the use of e-mental health in psychotherapy, as e-mental health tools were already used before the pandemic [1–3]. At least since the COVID-19 pandemic, a variety of modern technology-based interventions have been available to support mental health services. However, there is no common definition of e-mental health. Based on current research, we define e-mental health as all mental and behavioral health services that are delivered through a non-face-to-face setting via distance communication technologies such as videoconferencing, synchronous or asynchronous chats, or internet- or mobile-based interventions [4,5]. During the COVID-19 pandemic, psychotherapists had to use e-mental health to continue their treatment and stay in contact with patients [2,3,6]. Even after the pandemic, the majority of psychotherapists continued to use modern technologies such as videoconferencing. Most of the psychotherapists at least heard about e-mental health interventions [4]. However, the pandemic did not lead to increased use of all types of e-mental health. While videoconferencing in psychotherapy is here to stay [6–8], mental health apps (MHAs), chatbots or virtual reality continue to be rarely used by psychotherapists [4,8,9]. In a German online survey, only 29% of the participating mental health professionals reported having already used e-mental health in their practice [4]. Although psychotherapists generally have a positive attitude toward e-mental health, they are very reluctant to use it in their therapeutic work [10]. This leads to the following question: what are the reasons for the low use of e-mental health services?

Previous research has shown that the use of e-mental health in psychotherapy is beneficial and effective [11–14]. In particular, blended treatments have shown effect sizes comparable to those of face-to-face therapy [11,15–17]. Hedman-Lagerlöf et al. (2023) reported in their review about the effectiveness of psychotherapist-supported internet-based CBT that there were no differences in the effectiveness of interventions between different psychological disorders or between different severity levels. Furthermore, previous research has shown that e-mental health could increase the treatment adherence of patients [1,18,19]. Nevertheless, the perceptions and expectations of psychotherapists about the impact of e-mental health on psychotherapeutic treatments are different. Although psychotherapists know about the effectiveness of e-mental health treatments [20], they still think that evidence-based e-mental health could have a negative impact on clients’ engagement in therapy; therefore, e-mental health could have a negative impact on therapeutic outcomes [7]. The divergence between evidence-based effectiveness outcomes and the lasting doubts of psychotherapists might be explained by the lack of experience in e-mental health use. Little experience with e-mental health interventions was associated with more skeptical attitudes toward e-mental health and lower usage intentions of psychotherapists [20,21]. Moreover, the evidence-base of e-mental health tools is still unambiguous. Although there are a lot of effectiveness studies that pointed out the effectiveness of different e-mental health tools there are also results that did not show effectiveness of e-mental health tools [22]. Additionally, there is a high discrepancy between evidence-based and available e-mental health tools [23–25]. In result, psychotherapists had to be well informed to ensure using only evidence-based and beneficial e-mental health tools. Next to these uncertainties, also an expected increased workload lead psychotherapists to avoid changes in their work [6,10,21]. Before recommending e-mental health tools to patients or to use e-mental health tools in their psychotherapies, psychotherapists had to learn how to use e-mental health tools and must determine what kind of e-mental health intervention is suitable for their patients.

Another indicator for the extent of e-mental health use could be the psychotherapeutic orientation and working methods. In the European survey by Schuster et al. [26], the psychotherapeutic approach emerged as the second strongest variable for positive attitudes of psychotherapists. Research has shown that cognitive behavioral psychotherapists (CBTs) are more likely to use e-mental health interventions. Psychodynamic or psychoanalytic psychotherapists were less comfortable with e-mental health [20,26–28]. As a large proportion of e-mental health is based on CBT [29,30], this could represent a barrier to use for psychotherapists from other psychotherapeutic approaches and could result in less experience with e-mental health for non-CBT psychotherapists.

Previous research on psychotherapists’ e-mental health use has focused mainly on attitudes towards e-mental health and possible associations with their e-mental health use [6,26,31]. However, from the clients’ perspective one of the most important barriers is access to technology and the internet [20]. To our knowledge, to date, no studies have investigated the accessibility and availability of technology from psychotherapists’ perspectives. It can be assumed that technical barriers do not exclusively exist on the clients’ side. To understand potential associations between the availability of technology and psychotherapists use of e-mental health tools, it is essential to know what kind of technology is available at the psychotherapists’ workplace.

**Fig 1:**
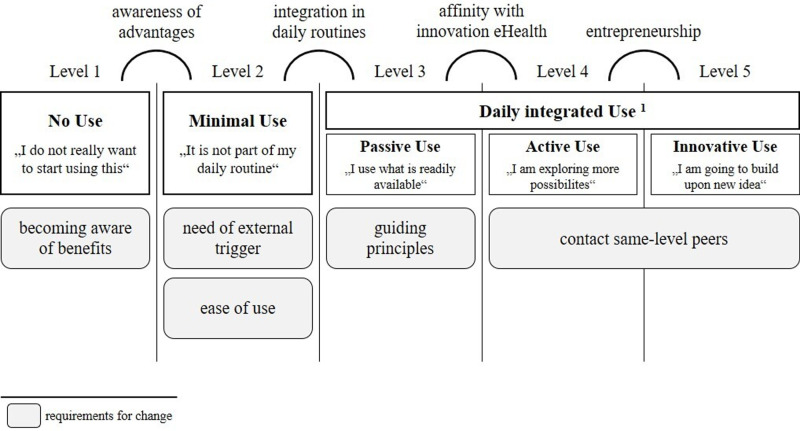
Adapted model of Levels of Adoption of eMental Health (LAMH). adapted from Feijt et al. (2018) presenting different Levels of e-mental health use depending on specific e-mental health tools and requirements for change according to the different Levels of use. Adaptations are marked with 1 in the illustration.

To gain an overview of potential influencing factors on general e-mental health use by psychotherapists, Feijt et al. developed the Levels of Adoption of e-mental health model (LAMH). Therein, the authors categorized the extent of e-mental health use into five different levels from Level 1 (no use) to Level 5 (innovative use) whereby the Levels 3 to 5 described different graduations of a daily integrated e-mental health use. Following the LAMH model, psychotherapists perceive different drivers, barriers and requirements for change for their e-mental health use according to the different levels of use. For example, having concrete guiding principles is no requirement for start using e-mental health tools. In the beginning (Level 1) psychotherapists had to become aware of benefits to start using these tools. So, they need a positive attitude toward e-mental health. Also, the role of the psychotherapeutic approach or technical equipment available at work can be assigned to the LAMH model. Both aspects could be indicators for becoming aware of benefits on the one hand or for perceived ease of use (requirement of change in Level 2) on the other hand. The adapted version of the LAMH model is presented in Fig 1. To better understand the needs of psychotherapists, we need to know their usage behavior first. Their needs could vary depending on their level of use.

### 1.1. Objectives

To understand which variables are associated with e-mental health use, we need to obtain a picture of the status quo of media equipment and use in the post-COVID-19 era. What kind of technical equipment is available at the psychotherapist’s workplace, and to what extent are modern technologies already part of the work routines of psychotherapists? The present study aims to provide some insight into this matter. Furthermore, the aim of the present study is to assess the usage of e-mental health after the COVID-19 pandemic and potential associations with their e-mental health usage in a sample of German psychotherapists. We examined the association between e-mental health use and the psychotherapeutic approach, attitudes toward the integration of e-mental health and the technical equipment available at the workplace. We hypothesized that the therapeutic range of use of e-mental health is associated with all three factors. Based on previous research [20,26], we expected that psychotherapists working with CBT would use a wider range of e-mental health in their psychotherapy than those who identify with a psychodynamic approach. Furthermore, we hypothesized that a high perceived potential to augment psychotherapy by using e-mental health and large amounts of technical equipment are associated with a wide range of use of e-mental health. Additionally, we predicted that high perceived risks of the use of e-mental health will be negatively associated with the range of use. Moreover, we focused on the subsample of psychotherapists who used MHAs in psychotherapy. Within this subsample, we examined the associations between the use of MHAs and psychotherapists’ attitudes toward the integration of e-mental health and the technical equipment available at the workplace. We predicted that the perceived potential to augment psychotherapy and the technical equipment available at the workplace are positively related to the use of MHAs. Perceived risks of e-mental health, on the other hand, were expected to correlate negatively with the use.

## 2. Methods

The study was an online survey study with a convenience sample of German psychotherapists that included all psychotherapeutic approaches.

### 2.1. Participants

The participants were 159 German psychotherapists who were licensed or in training. In Germany, psychotherapists complete a postgraduate training (3 to 5 years) following their master’s degree in psychology to obtain an official license to practice as psychotherapists. During postgraduate training, psychotherapists already work with patients. Thirty-nine additional respondents began the survey but dropped out before completing the first quarter of the survey. The participants were, on average, 44.02 years of age (*SD*=13.18). Almost half of the participants mainly treated children and adolescents (54%, *n*=86). The sample included a variety of psychotherapeutic approaches, but the majority identified with CBT (74%, *n*=117). Twenty-five percent of the psychotherapists drew on psychodynamic therapies and psychoanalysis (*n*=39). Only two people were systemic psychotherapists, and one reported belonging to another psychotherapeutic approach. The surplus of CBT therapists is representative of the distribution in Germany [32].

### 2.2. Procedure

The data was collected from 9^th^ January 2023 to 31^st^ March 2023 via LimeSurvey, an open source survey tool by Limesurvey GmbH [33]. The survey link was distributed via email through professional networks (e.g., psychotherapeutic working groups, psychotherapeutic associations, outpatient clinics, training institutes) and social media. At the beginning of the survey, participants received information about the study, the given anonymity, and voluntary nature of their participation. They had to give their written informed consent before continuing to the questions. It took approximately 15 minutes to complete the survey. The study was approved by the ethics review board of Bielefeld University.

### 2.3. Measures

The survey consisted of 44 questions about the primary psychotherapeutic approach, treating minors or adults, attitudes toward e-mental health and the individual use of modern technologies and e-mental health in private and professional contexts (see S1 File).

#### 2.3.1. Individual use of modern technologies and e-mental health.

Individual use of modern technologies in general was measured with purpose-built questions about technical equipment available at home or at work and utilization periods of modern technologies differentiating between private and professional use. To gather more detailed information about therapeutic integration of modern technologies, we asked questions about the consideration of clients’ media consumption in diagnostics, as well as the type and frequency of the use of videoconferencing and recommendations of MHAs in psychotherapy. In Germany, MHAs can be divided into digital healthcare applications (DiGAs), which can be officially prescribed by psychotherapists and physicians, and free MHAs. Both were covered in the survey. Certified DiGAs ensure the effectiveness and high data protection standards of the intervention. The costs of the apps were covered by health insurance, when the app was officially prescribed. DiGAs were available for a variety of psychological disorders of adults. Up to now, there are no DiGAs available for children. Because DiGAs are special to Germany, we summarized the items about DiGAs and free MHAs to one score for the statistical inference analyses. Therefore, we used the higher value of the two original variables for the merged variable. Questions about the frequency of use were answered on a six-point rating scale from 1 (once a year) to 6 (several times a week). The ownership of typical devices such as smartphones, tablets and computers was measured with dichotomous items (yes/no). To summarize the technical equipment available at work, a sum score was calculated for the three technical equipment items (smartphone, computer, and tablet) with a range from 0 to 3. To operationalize the therapeutic range of e-mental health use, the sum score of the dichotomous variables using videoconferencing at work, video therapy, free MHA and DiGAs was calculated. The potential sum score ranged from 0 to 2. Psychotherapists were asked to answer all questions about the use of modern technologies and e-mental health concerning the past six months.

#### 2.3.2. The psychotherapists’ attitudes toward using modern technologies in psychotherapy and Counselling Scale (MTPS).

The psychotherapists’ attitudes toward using modern technologies in psychotherapy and counselling scale [34] is a 16-item questionnaire with four subscales: (1) *potential to augment psychotherapy*, (2) *psychoeducational value*, (3) *perceived risks*, and (4) *perceived relevance*. The authors [34] defined modern technologies as general term for computers, smartphones, other technical equipment, video and audio materials, websites, e-books and mobile applications that are used for client communication or therapeutic methods within or between therapy sessions. Due to the current research question, we used the two subscales *potential to augment psychotherapy* and *perceived risks*. Both scales were identified as central factors of practitioners’ attitudes toward technology [27,34]. Psychoeducational features are not part of all e-mental health tools; therefore, we excluded the subscale *psychoeducational value* from the analyses. Furthermore, the subscale *perceived relevance* was not considered due to the similarity to the subscale *potential to augment psychotherapy*. The subscale *potential to augment psychotherapy* described the perception of practitioners that modern technology can increase the effectiveness of psychotherapy. The subscale *perceived risks* focused on practitioners’ concerns about security factors and the potential harmfulness of the therapeutic process by using modern technologies in psychotherapy. Participants rated different statements about their attitudes on a 5-point-rating-scale ranging from 1 (strongly disagree) to 5 (strongly agree). Higher scores represent more positive attitudes toward the psychotherapeutic use of modern technologies. Subscale scores are formed by the mean values of the four associated items of each subscale ranging from 1 to 5. For the current study, the MTPS was translated to German. Quality of translation was assured via multiple forward and backward translation. In the current study the internal consistency of the subscale *potential to augment* was comparable to that of previous studies, with Cronbach’s α=.86 (Bagarić and Jokić-Begić (2020): α=.86) [34]. The internal consistency of the subscale *perceived risks* was lower than that in previous studies, with Cronbach’s α=.56 (Bagarić and Jokić-Begić (2020): α=.62) [34].

### 2.4. Statistical analysis

The data was analyzed using IBM SPSS Statistics (Version 29). For analyses, missing values were deleted pairwise. The resulting differences in sample sizes are indicated in the respective analyses. In a first step, we categorized psychotherapists’ Levels of use according to the adapted LAMH model. Based on previous research results that showed that psychotherapists’ e-mental health use differed between different tools, we categorized use separately for different e-mental health tools. For e-mental health use, we focused on the use of videoconferencing and MHAs in the current article. The categorization into the adapted version of levels of use of e-mental health tools was done based on the frequency of use of the e-mental health tools. Psychotherapists who never used one e-mental health tool were categorized to Level 1 (no use), using e-mental health tools once a month or less were categorized into Level 2 (minimal use) and psychotherapists who used an e-mental health tool weekly were categorized into Level 3-5 (daily integrated e-mental health use). In a second step, we analyzed the associations of the variables *psychotherapeutic approaches*, the two scales *potential to augment psychotherapy* and *perceived risks* of the MTPS, and t*echnical equipment available at work* on the outcome *therapeutic range of e-mental health use* via sequential multiple linear regression. Because of the small subsample size of systemic therapy and other psychotherapeutic approaches (*n*=3), we excluded these participants from the regression analysis and converted the variable psychotherapeutic approaches into a dichotomous variable with the categories *psychodynamic therapy and psychoanalysis* and *cognitive behavioral therapy*. All preconditions were met. Linearity and homoscedasticity were analyzed via visual inspection of the scatterplots. For multicollinearity, we analyzed the variance inflation factor. We calculated a statistical power analysis for the planned analyses with *f*^2^=.15^,^ *α*=.05, β=.95. The statistical power was sufficient to identify small to moderate effects regarding the hypotheses. The analysis used a two-tailed alpha of *α*=.05. The strength of the variance explained by the coefficient of determination is interpreted according to Cohen (1988): small variance explanation *R*²=.02, medium variance explanation *R*²=.13, and large variance explanation *R*²=.26 [35]. For the regression, our metric for a small effect size was *f*^2^>.02, for a medium effect was *f*^2^>.15, and for a large effect was *f*^2^>.35 [36].

To test the relationship between the frequency of use of MHA recommendations and the two scales *potential to augment psychotherapy* and *perceived risks* of the MTPS as well as *the technical equipment available at work* within the subsample of psychotherapists who used MHAs, we used Spearman’s rank correlation coefficients. According to the hypotheses, all correlations were tested one-tailed. The convention for a small effect was |*r*|=.10, for a medium effect was |*r*|=.30, and for a large effect was |*r*|=.50 [35]. Bonferroni‒Holm corrections were applied to control for type-1 error accumulation. Only one psychotherapist with a psychotherapeutic approach other than CBT answered the question about the frequency of e-mental health use, wherefore associations between the psychotherapeutic approach and the frequency of use of MHAs could not be tested.

## 3. Results

### 3.1. Psychotherapists’ use of modern technologies

**Fig 2:**
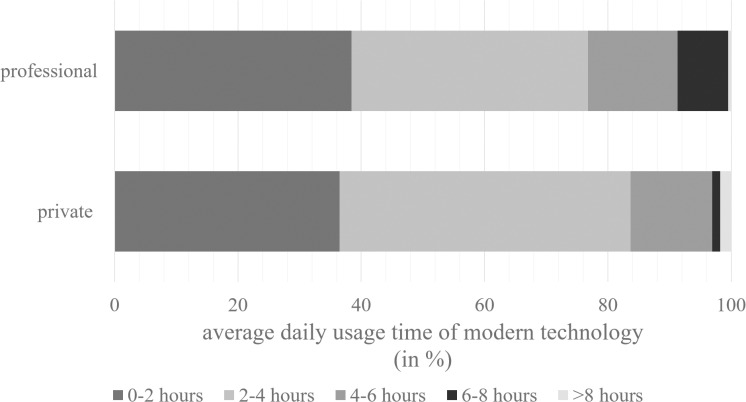
Distribution of the average usage times of modern technologies of psychotherapists divided into private and professional contexts (in percent).

The ownership of typical devices (smartphones, computers, and tablets) varied between private and professional contexts. All psychotherapists privately owned a smartphone and a laptop or computer. In contrast, only 65% of the psychotherapists (*n*=103) used a smartphone at work, and 93% of the psychotherapists (*n*=148) had a computer or a laptop at work. While 60% of the psychotherapists (*n*=94) owned a tablet privately, only 25% of the psychotherapists owned a tablet for professional context (*n*=39). The results of the daily usage time of modern technologies at home and at work are presented in Fig 2. Almost half of the psychotherapists used messenger apps at work (48%, *n*=76). Social media and streaming applications were used less frequently, with 9% of the psychotherapists using social media and 23% using streaming applications at work. However, 124 out of 159 psychotherapists (78%) used videoconferencing at work, and 120 out of 159 psychotherapists performed video therapy (76%). Forty-five percent of the psychotherapists who used video therapy for their sessions reported that they had offered video therapy to more than ten patients in the past six months. Free MHAs were recommended by 17% of the psychotherapists (*n*=27), 37% of whom recommended MHAs to more than ten patients in the past six months. DiGAs were prescribed by eight psychotherapists (5%), with two of them prescribing DiGAs to more than ten patients in the past six months. The results of the frequency of use of video therapy, MHAs and DiGAs are presented in Table 1. In general, 26 out of 159 psychotherapists (16%) always enquired patients’ personal media use in the diagnostics, 37 psychotherapists (23%) often, 65 psychotherapists (41%) sometimes and 31 psychotherapists (20%) reported never enquiring it. No difference between psychotherapists treating minors and psychotherapists treating adults was found concerning e-mental health use (χ²(2)=1.49, *p*=.475). This differentiation was therefore not taken into account further.

**Table 1:** Frequency of use of e-mental health in psychotherapy.

		Video therapy	Freely available mental health apps	Digital health care applications (DiGAs)
Usage in total^a^		120 (76%)	27 (17%)	8 (5%)
	Several times a week	40 (25%)	1 (1%)	0 (0%)
	Once a week	33 (21%)	5 (3%)	1 (1%)
	Once a month	27 (17%)	10 (6%)	2 (1%)
	Once in a quarter year	12 (8%)	10 (6%)	2 (1%)
	Once in a half year	8 (5%)	1 (1%)	1 (1%)
No use^b^		38 (24%)	130 (82%)	149 (94%) ^c^

^a^number of participants who reported to use the e-mental health tools,

^b^number of participants who reported to not use the e-mental health tools,

^c^DiGAs are not available for treating minors, therefore psychotherapists who were mainly treating minors were not able to recommend DiGAs (*n*=86)

Psychotherapists use of e-mental health differed between videoconferencing tools and MHAs. For MHAs, 130 out of 157 psychotherapists were categorized into Level 1 of the adapted LAMH model with not using MHAs in psychotherapeutic contexts, 21 participants were categorized in Level 2 (minimal use) and solely 6 participants were categorized in Level 3 by integrating MHAs in daily work routines. In contrast, for videoconferencing use, the majority of psychotherapists were categorized in level 2 with 73 psychotherapists out of 159 (46%) using videoconferencing at least once a week. 47 psychotherapists showed minimal videoconferencing use (Level 2) and 38 psychotherapists (24%) never used videoconferencing (Level 1).

### 3.2. E-mental health use and psychotherapeutic approaches, attitudes toward modern technologies and technical equipment available at work

The first regression model with the psychotherapeutic approach as a variable explained 5% of the variance in the range of therapeutic use of modern technologies (*R*^2^=.05, *F*(1,146)=3.08, *p*=.006, *f*^*2*^=0.05). Adding the two scales *potential to augment psychotherapy* and *perceived risks* of the MTPS as additional variables improved the model significantly (Δ*R*^*2*^ =.06, *F*(3,144)=6.74, *p*<.001, *f*^*2*^=0.12). In the third step, adding *technical equipment available at work* as an additional variable improved the model again significantly (Δ*R2* =.05, *F*(4,143)=9.97, *p*<.001, *f*^*2*^=0.19). As shown in Table 2, *potential to augment psychotherapy* and *technical equipment available at work* were positively related to the range of therapeutic use of e-mental health. *Perceived risks* were negatively related to the range of implemented e-mental health. The total regression model explained 16% of the variability in the range of therapeutic use of e-mental health. The variance explained by the regression model was moderate. The descriptive statistics of the relevant variables of the sequential multiple regression are presented in Table 3.

**Table 2:** Linear model of predictors of therapeutic range of modern technology use. 95% bias corrected and accelerated confidence intervals reported in parentheses.

		Range of therapeutic use of modern technologies			
Predictor variables		*B*	*SE B*	β	*p*
Step 1					
	constant	0.84 (0.64, 1.05)	0.10		<.001
	Psychotherapeutic approach	0.33 (0.10, 0.57)	0.12	0.22	.006
Step 2					
	constant	0.55 (-0.12, 1.21)	0.34		.107
	Psychotherapeutic approach	0.22 (-0.03, 0.46)	0.13	0.15	.087
	Potential to augment psychotherapy	0.20 (0.05, 0.34)	0.07	0.23	.009
	Perceived risks	-0.10 (-0.24, 0.04)	0.07	-0.12	.142
Step 3					
	constant	-0.02 (-0.76, 0.73)	0.38		.969
	Psychotherapeutic approach	0.26 (0.02, 0.50)	0.12	0.17	.037
	Potential to augment psychotherapy	0.23 (0.09, 0.37)	0.07	0.26	.002
	Perceived risks	-0.09 (-0.22, 0.05)	0.07	-0.10	.197
	Technical equipment available at work	0.19 (0.07, 0.32)	0.07	0.24	.003

*n*=148; *R*^2^=.05 (*p*=.006) for step 1; Δ*R*^2^=.06 (*p*=.010) for step 2; Δ*R*^2^=.05 (*p=*.003) for step 3.

**Table 3:** Descriptive statistics of all relevant interval-scaled variables for the sequential multiple regression.

Variable	*n*	*M*	*SD*	Range
Range of therapeutic use of e-mental health	148	1.09	0.65	0-2
Potential to augment psychotherapy	148	3.62	0.75	1-5
Perceived risks	148	3.16	0.73	1.25-5
Technical equipment available at work	148	1.86	0.79	0-3

*M*=mean, *SD*=standard deviation.

Spearman’s rank correlation was computed to assess the relationship between the frequency of use of MHA recommendations and the two scales *potential to augment psychotherapy* and *perceived risks* as well as the *technical equipment available at work* within the subsample of psychotherapists who used MHAs (*n*=27). The frequency of MHA recommendation and *potential to augment psychotherapy* were not significantly correlated (*r*=.06, *p*=.380; after the Bonferroni–Holm correction, *p*=.380). After adjusting for alpha-inflation, *technical equipment at work* (*r*=.36, *p*=.031, after Bonferroni–Holm correction *p*=.062) and *perceived risks* (*r*=-.41, *p*=.018, after Bonferroni–Holm correction *p*=.054) did not significantly correlate with the frequency of MHA recommendations. The descriptive statistics of the relevant variables of the Spearman-ranked correlations are presented in Table 4.

**Table 4:** Descriptive statistics of all relevant interval-scaled variables for the Spearman’s rank correlations.

Variable	*n*	*M*	*SD*	Range
Frequency of use of mental health apps	27 ^a^	3.93	0.87	3-6
Potential to augment psychotherapy	29	4.03	0.45	3-5
Perceived risks	29	2.87	0.75	1.25-5
Technical equipment available at work	29	1.86	0.88	0-3

*M*=mean, *SD*=standard deviation.

^a^Two psychotherapists reported to use mental health apps but did not answer the question about their frequency of use.

## 4. Discussion

The objective of the current study was to assess the usage of and potential association factors with e-mental health in a sample of German psychotherapists. The available technical equipment varied between work and home. Psychotherapists had limited equipment available at work. Moreover, psychotherapists used little e-mental health. Solely videoconferencing was used in psychotherapies regularly. Following the LAMH model, psychotherapists’ use of videoconferencing could be mainly categorized into Levels 3-5 (daily integrated e-mental health use). In contrast, according to their use of MHAs, most of the psychotherapists had to be categorized into Level 1 (no use). The psychotherapeutic approach, potential to augment psychotherapy and technical equipment available at work were significantly associated to the therapeutic range of use of e-mental health as hypothesized. However, the frequency of use of MHAs did not show significant associations with the potential to augment psychotherapy, perceived risks, or technical equipment available at work after adjusting for alpha inflation. Nevertheless, small trends were recognizable for the associations between the use of MHAs and perceived risks and between the use and the amount of technical equipment available at work.

Digital services such as streaming applications or social media were used very seldomly. In the current sample, less than half of the psychotherapists regularly enquired the media use by patients. Correspondingly, the utilization of MHAs is far from being integrated into daily routines. With 82% of the psychotherapists not recommending MHAs in psychotherapy, most of the psychotherapists were still at Level 1 concerning the use of MHAs. Looking at the 27 psychotherapists who reported using MHAs for psychotherapies, 21 out of the 27 psychotherapists could be rated as Level 2 with the occasional use of MHAs. Only six psychotherapists can be categorized at Level 3 of the LAMH, recommending MHAs at least once a week. In line with previous research, the use of video therapy drew a different picture in the categorization of its Levels of use [4,6–8]. One hundred and twenty out of 158 psychotherapists used video therapy, and half of the psychotherapists reported offering video therapy at least once a week. These psychotherapists can be categorized at Level 3 (passive use) of the LAMH for video therapy. Furthermore, 30% of the psychotherapists reported using video therapy occasionally. These psychotherapists can be categorized on Level 2 of the LAMH. Regarding the LAMH model, an explanation of the differences between the Levels of use of video therapy and MHAs might be that the COVID-19 pandemic contention measures worked as an external trigger for implementing video therapy. During the phases of social distancing, psychotherapists had to use video therapy to continue their psychotherapy [2,6]. During this period, psychotherapists were able to have positive experiences with video therapy and became accustomed to this way of working [21]. As Békés and Aafjes-van Doorn [21] noted, psychotherapists experienced mandatory change as positive and useful for their work. Consequently, an external trigger might be essential for integrating e-mental health into the daily routines of psychotherapists. This is in line with Level 2 of the LAMH model: a change requires external triggers.

To answer the question of what is necessary to start using e-mental health, “becoming aware of benefits” is one important requirement of Level 1 users according to the LAMH model. The potential to augment psychotherapy, an important part of the benefits of e-mental health, was a significant variable of the therapeutic range of e-mental health use. Within the subsample of psychotherapists who used MHAs, the association between the potential to augment psychotherapy and the frequency of use of MHAs was not significant. Thus, being aware of the benefits of e-mental health is positively associated with the use of e-mental health (Level 1) but not with the use of MHA specifically (Levels 2 and 3 users).

Furthermore, technical equipment and skills in handling play a role in the perception of benefits: the more equipment is available at the psychotherapist’s workplace, the more e-mental health is used. The technical equipment available at work has received little attention in the literature to date. To the best of our knowledge, this is one of the first studies to assess what kind of technical equipment is available at psychotherapists’ workplaces. Therefore, available equipment can be a crucial barrier to the use of e-mental health. On the one hand, little experience in e-mental health interventions is associated with more skeptical attitudes toward e-mental health [20,21]. On the other hand, technical equipment and skills in handling are also basic prerequisites for the perceived ease of use of e-mental health [20,21]. More experience with technology can dismantle reservations and increase the perceived ease of use. If psychotherapists were familiar with technical equipment and e-mental health in general, it would be easier for them to start using new e-mental health tools as well as to integrate them into their daily routines [20,21]. In line with these findings, the amount of technical equipment available at work was not only associated of the therapeutic range of use.

Moreover, the fit of e-mental health content to the therapeutic approach may contribute to the perceived ease of use. In total, 93% of the psychotherapists who recommended MHAs or DiGAs worked with CBT. Correspondingly, most of the available e-mental health interventions are CBT-related interventions [29,30]. Hence, these CBT-based interventions may be advantageous for psychotherapists with a corresponding psychotherapeutic approach.

In the subsample of psychotherapists who used MHAs at least occasionally, risk perception gained importance. Although the negative association between perceived risks and the frequency of use of MHAs was not significant, some trends were detected even in the small sample of 27 psychotherapists. Surprisingly, DiGAs had extremely low usage rates, with only 8 out of 158 psychotherapists prescribing DiGAs. Certified DiGAs ensure the effectiveness and high data protection standards of the intervention. According to the LAMH, these security standards should address psychotherapists’ need for guiding principles at Level 3. However, the use of DiGAs was even lower than the use of free MHAs. One barrier to the use of DiGA might be its limited availability for adults. There are no DiGAs available for children and adolescents, which excludes a large target group. Furthermore, these results could lead to the assumption that in addition to perceived risks of use, other variables are important for using e-mental health regularly. Further correlates of e-mental health use at Level 3 should be examined in future studies.

### 4.1. Practical implications and future research

E-mental health has the potential to address the escalating treatment burden in the healthcare system since the COVID-19 pandemic [1,3]. Utilizing MHAs can reduce waiting periods and allow individuals to receive treatment earlier. The present study underlines the importance of differentiating between the Levels of e-mental health use in psychotherapy in research and implementation strategies. Level 1 e-mental health users have different needs than Level 3 users. To start using e-mental health, psychotherapists must be convinced of the benefits of e-mental health for their psychotherapeutic work. Only subsequently did the ease of use or the necessity for guiding principles become relevant for the integration of e-mental health into the daily routines of psychotherapists. Moreover, the study results showed indications of the need for an external trigger for the integration of e-mental health tools. In contrast to MHAs, video therapy has become an integral part of psychotherapy due to the pandemic. There is an urgent need to focus on implementation approaches for nonusers of e-mental health, as they make up the vast majority of psychotherapists. Future research is required to examine the barriers and drivers to using e-mental health and to address the discrepancy between the evidence-based effectiveness of e-mental health and the remaining skepticism toward it. If we understand the relevant variables for e-mental health use in Level 1 users, it is possible to create more targeted offers and support possibilities for this group and to enable the change from nonusers to e-mental health users. In addition, future research should combine psychotherapists’ and patients’ perspectives and characteristics to analyze the use of e-mental health. On the one hand, psychotherapists are the gatekeepers for patients’ e-mental health use. On the other hand, e-mental health tools need to fit to patients’ needs as well as make them use it.

### 4.2. Strengths and limitations

Overall, the current study was an important step in obtaining initial insights into the variables associated with e-mental health use in psychotherapy for different user types. However, there is still a large fraction in the variability of therapeutic use unexplained by the model. Potentially, other important factors were not considered in the model. Aforementioned, future research should address further potential association factors as other personal characteristics of psychotherapists (e.g., gender, age) or patients’ characteristics. To the best of our knowledge, this is one of the first studies assessing the technical equipment available at work from psychotherapists and linking the technical equipment available at work with the e-mental health use of psychotherapists. However, only the ownership of smartphones, computers, and tablets was assessed in the current study to operationalized the technical equipment available at work. In future research it should be considered to assess the technical equipment available at work in more detail. Furthermore, the current study focused on the use of videoconferencing and MHAs. Another limitation was the convenience sample and small sample size with 158 German psychotherapists. The current study was an anonymous online survey to mitigate potential response bias. Nevertheless, the possibility of potential response bias in self-reports and potential selection bias due to the study design cannot be excluded. Regarding to these limitations, the findings may not be generalized to all psychotherapists, countries or all types of e-mental health interventions. Therefore, further research is necessary. Additionally, the cross-sectional design does not allow any conclusions about causality.

## 5. Conclusion

The findings of the current study, suggest that the use of e-mental health was not well integrated into the daily working routines of psychotherapists. Furthermore, many psychotherapists had limited technical equipment available at workplace. The technical equipment, attitudes toward e-mental health, psychotherapeutic approach, and external trigger emerged as potentially important variables for starting the implementation of e-mental health. To address low e-mental health use, it is necessary to better understand the correlates of e-mental health use at different Levels of use. Therefore, further research within a larger, representative sample of psychotherapists is important.

## Supporting information

S1 File List of questions included in the Online-Survey.
The original survey was in German. For this publication, the survey was translated in English.
(PDF)

S2 File Data set.
(CSV)

S3 File Data dictionary of the data set.
(CSV)
