# Supplementary material for: Why do psychotherapists use so little e-mental health in psychotherapy? Insights from a sample of psychotherapists in Germany
Source: PLOS Ment Health. 2025 Mar 19;2(3):e0000270. doi: 10.1371/journal.pmen.0000270 (PMC12798207; doi:10.1371/journal.pmen.0000270)
Supplement: S1 File — The original survey was in German. For this publication, the survey was translated in English. (PDF) [file pmen.0000270.s001.pdf]

S1 File: List of questions included in the Online-Survey

The original survey was in German. For this publication, the survey was translated in English.

Soziodemografie / Sociodemographics
(selbst erstellte Items) / (self-created items)

Zunächst bitten wir Sie um einige Angaben zu Ihrer Person. / First of alll, please provide us with some personal details.

Wie alt sind Sie? / How old are you?

____

Nach welcher Therapieschule arbeiten Sie hauptsächlich? / Which psychotherapeutic approach do you mainly identify with?

- ☐ Verhaltenstherapie / Cognitive Behavioral therapy
- ☐ Tiefenpsychologische Therapie / Psychodynamic therapy
- ☐ Analytische Therapie / Psychoanalysis
- ☐ Systemische Therapie / Systemic therapy
- ☐ Anderes / Other

  Nach welcher Therapieschule unterrichten Sie dann? / Which psychotherapeutic approach do you identify with?

  ____

Ich behandle hauptsächlich... / I mainly treat...

- ☐ Erwachsene / Adults
- ☐ Kinder und Jugendliche / Children and adolescents

Medienaffinität: Besitz und Nutzung digitaler Medien
Media affinity: Ownership and use of digital media
(selbst erstellte Items) / (self-created items)

Im Folgenden möchten wir von Ihnen wissen, ob, welche und wie Sie neue Medien verwenden. Da sich das Mediennutzungsverhalten im privaten und beruflichen Kontext stark unterscheiden kann, möchten wir gern mehr über beide Bereiche erfahren. Die folgenden Fragen beziehen sich auf das letzte halbe Jahr.
In the following, we would like to learn from you whether, which, and how you use new media. As media usage behavior can differ greatly in private and professional contexts, we

would like to find out more about both areas. The following questions relate to the last six months.

Zunächst möchten wir mehr über Ihre Nutzung neuer Medien im **privaten** Kontext erfahren. / First of all, we would like to find out more about your use of new media in a **private** context.

Privat: Besitzen Sie ein Smartphone? / Private: Do you own a smartphone?

- ☐ Ja / Yes
- ☐ Nein / No

Privat: Besitzen Sie einen Laptop/Computer? / Private: Do you own a laptop/computer?

- ☐ Ja / Yes
- ☐ Nein / No

Privat: Besitzen Sie ein Tablet? / Private: Do you own a tablet?

- ☐ Ja / Yes
- ☐ Nein / No

Privat: Wie viel Zeit am Tag nutzen Sie durchschnittlich Ihr Smartphone/Computer/ Laptop/Tablet? / Private: How much time a day do you use your smartphone/computer/ laptop/tablet on average?

- ☐ 0-2 Stunden / Hours
- ☐ 2-4 Stunden / Hours
- ☐ 4-6 Stunden / Hours
- ☐ 6-8 Stunden / Hours
- ☐ mehr als 8 Stunden / Hours

Privat: Wie viel Zeit davon nutzen Sie neue Medien (Smartphone/Computer/Laptop/Tablet) durchschnittlich... (in %) / Private: How much time do you use new media (smartphone/computer/laptop/tablet) on average... (in %)

- zum Telefonieren? / to make phone calls? ____
- zum Chatten? / to chat? ____
- für Social Media (z.B. Instagram)? / for social media (e.g. Instagram)? ____
- für YouTube? / for YouTube? ____
- für Spiele? / for games? ____
- für Anderes? / for other things? ____

Für was nutzen Sie ihr Smartphone/Computer/Laptop/Tablet noch? / What else do you use your smartphone/computer/laptop/tablet for?

____

Privat: Haben Sie bereits an Videokonferenzen teilgenommen? / Private: Have you already taken part in video conferences?

- ☐ Ja / Yes
- ☐ Nein / No

Privat: Wie häufig nehmen Sie an Videokonferenzen teil? / Private: How often do you take part in video conferences?

- ☐ Mehrmals in der Woche / Several times a week
- ☐ Einmal in der Woche / Once a week
- ☐ Einmal im Monat / Once a month
- ☐ Einmal im Quartal / Once a quarter
- ☐ Einmal im halben Jahr / Once every six months
- ☐ Einmal im Jahr / Once a year

Privat: Welche der folgenden Apps nutzen Sie? Sie können mehrere Apps auswählen. / Private: Which of the following apps do you use? You can select several apps.

- ☐ WhatsApp
- ☐ Telegram
- ☐ Signal
- ☐ Threema
- ☐ andere Messenger-App / other messenger app
- ☐ Instagram
- ☐ Snapchat
- ☐ Twitter
- ☐ YouTube
- ☐ TikTok
- ☐ Facebook
- ☐ Spotify
- ☐ Pinterest
- ☐ Andere / other

  Welche anderen Apps verwenden Sie? / What other apps do you use?

  ____

  ____

Beruflich: Besitzen Sie ein Smartphone? / Professionally: Do you own a smartphone?

- ☐ Ja / Yes
- ☐ Nein / No

Beruflich: Besitzen Sie einen Laptop/Computer? / Professional: Do you own a laptop/computer?

- ☐ Ja / Yes
- ☐ Nein / No

Beruflich: Besitzen Sie ein Tablet? / Professionally: Do you own a tablet?

- ☐ Ja / Yes
- ☐ Nein / No

Beruflich: Wie viel Zeit am Tag nutzen Sie durchschnittlich Ihr Smartphone/Computer/ Laptop/Tablet? / Professionally: How much time a day do you use your smartphone/ computer/laptop/tablet on average?

- ☐ 0-2 Stunden / Hours

- ☐ 2-4 Stunden / Hours
- ☐ 4-6 Stunden / Hours
- ☐ 6-8 Stunden / Hours
- ☐ mehr als 8 Stunden / Hours

Beruflich: Wie viel Zeit davon nutzen Sie neue Medien (Smartphone/Computer/Laptop/Tablet) durchschnittlich? (in %) / Professionally: How much time do you use new media (smartphone/computer/laptop/tablet) on average? (in %)

zum Telefonieren? / to make phone calls? ____
zum Chatten? / to chat? ____
für Social Media (z.B. Instagram)? / for social media (e.g. Instagram)? ____
für YouTube? / for YouTube? ____
für Spiele? / for games? ____
für Anderes? / for other things? ____

Für was nutzen Sie ihr Smartphone/Computer/Laptop/Tablet noch? / What else do you use your smartphone/computer/laptop/tablet for?

____
____

Beruflich: Haben Sie bereits an Videokonferenzen, außerhalb der Therapie, teilgenommen? / Professional: Have you already taken part in video conferences outside of therapy?

- ☐ Ja / Yes
- ☐ Nein / No

Beruflich: Wie häufig nehmen Sie an Videokonferenzen, teil? / Professionally: How often do you take part in video conferences?

- ☐ Mehrmals in der Woche / Several times a week
- ☐ Einmal in der Woche / Once a week
- ☐ Einmal im Monat / Once a month
- ☐ Einmal im Quartal / Once a quarter
- ☐ Einmal im halben Jahr / Once every six months
- ☐ Einmal im Jahr / Once a year

Beruflich: Welche der folgenden Apps nutzen Sie? Sie können mehrere Apps auswählen. / Professional: Which of the following apps do you use? You can select several apps.

- ☐ WhatsApp
- ☐ Telegram
- ☐ Signal
- ☐ Threema
- ☐ andere Messenger-App / other messenger app
- ☐ Instagram
- ☐ Snapchat
- ☐ Twitter
- ☐ YouTube
- ☐ TikTok

- ☐ Facebook
- ☐ Spotify
- ☐ Pinterest
- ☐ Andere / other

  Welche anderen Apps verwenden Sie? / What other apps do you use?

  ____

  ____

Beruflich: Erfassen Sie während der Probatorik Angaben zur Mediennutzung Ihrer Patient:innen? / Professional: Do you gather information on your patients' media use during diagnostics?

- ☐ Nie / Never
- ☐ Manchmal / Sometimes
- ☐ Häufig / often
- ☐ Immer / Always

Einsatz neuer Medien in der Psychotherapie
Use of new media in psychotherapy
(selbst erstellte Items) / (self-created items)

Anschließend möchten wir mehr über Ihren Einsatz von neuen Medien in der Psychotherapie erfahren. Auch diese Fragen beziehen sich auf das letzte halbe Jahr.
We would then like to find out more about your use of new media in psychotherapy.
These questions also relate to the last six months.

Nutzen Sie neue Medien während ihrer Sitzungen (z.B. Videokonferenzen, Apps, Youtube, Virtual Reality)? / Do you use new media during your meetings (e.g. video conferencing, apps, YouTube, virtual reality)?

- ☐ Ja / Yes
- ☐ Nein / No

  Welche neuen Medien nutzen sie? Sie können mehrere neue Medien auswählen. / Which new media do you use? You can select several new media.

  - ☐ Videokonferenzen / Video conferencing
  - ☐ Apps
  - ☐ YouTube
  - ☐ Virtual Reality
  - ☐ Anderes / Other

    Was verwenden Sie noch? / What else do you use?

    ____

Führen Sie Videotherapie durch? / Do you provide video therapy services?

- ☐ Ja / Yes
- ☐ Nein / No

Mit wie vielen Patient:innen haben Sie schon Videotherapie durchgeführt? Bitte geben Sie eine möglichst konkrete Anzahl an. / How many patients have you previously treated with video therapy? Please provide as specific as possible a number.

____

Wie häufig führen Sie Videotherapie durch? / How often do you provide video therapy services?

- ☐ Mehrmals in der Woche / Several times a week
- ☐ Einmal in der Woche / Once a week
- ☐ Einmal im Monat / Once a month
- ☐ Einmal im Quartal / Once a quarter
- ☐ Einmal im halben Jahr / Once every six months
- ☐ Einmal im Jahr / Once a year

Als nächstes möchten wir nun mehr über Ihren Einsatz von Apps in der Psychotherapie erfahren. Dabei geht es um Digitale Gesundheitsanwendungen und weitere Mental Health Apps. Die Fragen beziehen sich auf das letzte halbe Jahr.

Unter digitalen Gesundheitsanwendungen werden digitale Medizinprodukte niedriger Risikoklassen verstanden, die die Versicherten etwa bei der Behandlung von Erkrankungen oder dem Ausgleich von Beeinträchtigungen unterstützen können (DiGA, 2022). Digitale Gesundheitsanwendungen können von Psychotherapeut:innen oder Ärzt:innen verordnet und durch die Krankenkassen erstattet werden. In dieser Befragung geht es um die DiGAs, die sich auf die Psyche beziehen.
Next, we would like to find out more about your use of apps in psychotherapy. This is about digital healthcare applications and other mental health applications. The questions relate to the last six months.

Digital healthcare applications are understood to be digital medical devices of low risk classes that can support the insured person, for example in the treatment of illnesses or the compensation of impairments (DiGA, 2022). Digital healthcare applications can be prescribed by psychotherapists or doctors and reimbursed by health insurance companies. This survey focuses on the DiGAs that relate to mental health.

Verschreiben Sie digitale Gesundheitsanwendungen? (Ja/Nein) / Do you prescribe digital health applications? (Yes/No)

- ☐ Ja / Yes
- ☐ Nein / No

Welche der aufgeführten Apps haben Sie schon verschrieben? /
Which of the apps listed have you already prescribed?

- ☐ Deprexis
- ☐ edupression.com
- ☐ elona therapy Depression
- ☐ HelloBetter Panik / HelloBetter Panic
- ☐ HelloBetter ratiopharm chronischer Schmerz / HelloBetter ratiopharm chronic pain

- ☐ HelloBetter Schlafen / HelloBetter Sleep
- ☐ HelloBetter Vaginismus Plus
- ☐ Invirto – Die Therapie gegen Angst / Invirto - The therapy against anxiety
- ☐ Mindable: Panikstörung und Agoraphobie / Mindable: panic disorder and agoraphobia
- ☐ Selfapys Online-Kurs bei Binge-Eating-Störung / Selfapys online course for binge eating disorder
- ☐ Selfapys Online-Kurs bei Bulimia Nervosa / Selfapys online course for bulimia nervosa
- ☐ Selfapys Online-Kurs bei Depression / Selfapys online course for depression
- ☐ Selfapys Online-Kurs bei Generalisierten Angststörung / Selfapys online course for generalized anxiety disorder
- ☐ Somnio
- ☐ Velibra
- ☐ Vorvida

Wie vielen Patient:innen haben Sie schon digitale Gesundheitsanwendungen verschrieben? Bitte geben Sie eine möglichst konkrete Anzahl an. / How many patients have you already prescribed digital healthcare applications to? Please provide as specific as possible a number.

____

Wie häufig verschreiben Sie Digitale Gesundheitsanwendungen? / How often do you prescribe digital healthcare applications?

- ☐ Mehrmals in der Woche / Several times a week
- ☐ Einmal in der Woche / Once a week
- ☐ Einmal im Monat / Once a month
- ☐ Einmal im Quartal / Once a quarter
- ☐ Einmal im halben Jahr / Once every six months
- ☐ Einmal im Jahr / Once a year

Jetzt möchten wir mehr darüber erfahren, ob und welche "Mental Health Apps" Sie empfehlen. Mit „Mental Health Apps“ sind alle weiteren Apps und Webanwendungen gemeint, die die psychische Gesundheit von Patient:innen positiv beeinflussen, aber nicht unter die digitalen Gesundheitsanwendungen fallen. Sie werden zum Teil von den Krankenkassen finanziert (beispielsweise die App 7Mind), teilweise aber auch nicht (beispielsweise die App Headspace). Zudem sind hiermit auch die Online-Programme von Krankenkassen gemeint, die nur für die Mitglieder der spezifischen Krankenkasse zu Verfügung stehen. Die Unterstützung durch die Anwendung kann sehr unterschiedlich sein. Sie müssen sich nicht explizit auf die Behandlung einer Störung beziehen, sondern können sich beispielsweise auch auf andere Aspekte von psychischer Gesundheit fokussieren, wie Stressreduktion, Achtsamkeit, Stimmungschecks oder Schlafhygiene. Bitte beantworten Sie auch diese Fragen bezogen auf das letzte halbe Jahr.
Now we would like to find out more about whether and which mental health applications you recommend. "Mental health applications" refers to all other apps and web applications that positively influence the mental health of patients but do not fall under the category of digital healthcare applications. Some of them are funded by health insurance companies (e.g. the 7Mind app), but some are not (e.g. the Headspace app). This also includes the online

programs of health insurance companies, which are only available to members of the specific health insurance company. The support provided by the application can vary greatly. They do not have to relate explicitly to the treatment of a disorder but can also focus on other aspects of mental health, such as stress reduction, mindfulness, mood checks or sleep hygiene.
Please also answer these questions in relation to the last six months.

Nutzen Sie „Mental Health Apps" für Ihre Behandlung? / Do you use "Mental Health Applications" for your treatment?

- ☐ Ja / Yes
- ☐ Nein / No

Welche „Mental Health Apps" benutzen Sie? /
Which mental health applications do you use?

____

Wie vielen Patient:innen haben Sie schon „Mental Health Apps" empfohlen? Bitte geben Sie eine möglichst konkrete Anzahl an. / How many patients have you already recommended "Mental Health Applications" to? Please provide as specific as possible a number.

____

Wie häufig empfehlen Sie „Mental Health Apps"? / How often do you recommend "Mental Health Applications"?

- ☐ Mehrmals in der Woche / Several times a week
- ☐ Einmal in der Woche / Once a week
- ☐ Einmal im Monat / Once a month
- ☐ Einmal im Quartal / Once a quarter
- ☐ Einmal im halben Jahr / Once every six months
- ☐ Einmal im Jahr / Once a year

Einstellung zur Nutzung Moderner Technologien in der Psychotherapie

Attitude toward using modern technologies in Psychotherapy

MTPS: The Therapists´ Attitudes toward using Modern Technologies in Psychotherapy and Counselling Scale (Bagarić & Jokić-Begić, 2020).
Aus dem Englischen ins Deutsche übersetzt. / Translated from English into German.

Nun möchten wir von Ihnen wissen, wie Ihre Einstellung zu modernen Technologien ist. Der Begriff „moderne Technologien" bezieht sich auf: die Verwendung von PCs, Smartphones oder anderen Geräten; die Verwendung von Video- und Audiomaterialien, Webseiten, E-Büchern, Anwendungen und Programmen (z. B. Skype, Social Media, Viber, WhatsApp) für die Kommunikation mit Klienten, als Teil der therapeutischen Technik in der Sitzung oder als Teil der Aufgabe des Klienten zwischen den Sitzungen.
Now we would like to know what your attitudes are toward using modern technologies. The term "modern technologies" refers to: the use of PCs, smartphones or other devices; the use of video and audio materials, websites, e-books, applications and programs (e.g. Skype, social

media, Viber, WhatsApp) for communication with clients, as part of the therapeutic technique in session or as part of the client's task between sessions.

**Bitte geben Sie an, wie sehr Sie den folgenden Aussagen zustimmen. / Please indicate how strongly you agree with the following statements.**	Stimme gar nicht zu / strongly disagree	Stimme eher nicht zu / somewhat disagree	Weder noch / Neither agree nor disagree	Stimme eher zu / somewhat agree	Stimme voll und ganz zu / strongly agree
Moderne Technologien können den therapeutischen Prozess beschleunigen. / Modern technologies can speed up the therapeutic process.					
Moderne Technologien können die Wirksamkeit der Psychotherapie erhöhen. / Modern technologies can increase the effectiveness of psychotherapy.					
Moderne Technologien können zu Vorteilen für Klient:innen führen, die mit anderen Methoden nicht erreicht werden können. / Modern technologies can result in benefits for clients that cannot be achieved by other methods.					
Moderne Technologien können den therapeutischen Prozess bereichern. / Modern technologies can enrich the therapeutic process.					
Der Einsatz von modernen Technologien für die Psychoedukation kann nützlich sein (z. B. E-Books, E-Broschüren). / Using MTs for psychoeducation can be useful (e.g., e-books, e-brochures)					
Audio- und Videomaterial kann für therapeutische Zwecke nützlich sein. / Audio and video material can be useful for therapeutic purposes.					

Therapeut:innen sollten flexibel sein, was den Einsatz von modernen Technologien in der Psychotherapie angeht. / Therapists should be flexible regarding the use of modern technologies in psychotherapy.					
Multimediale Inhalte können bei einigen Klient:innen nützlich sein. / Multimedia content can be useful with some clients.					
Therapeut:innen sollten bei der Verwendung von modernen Technologien in der Psychotherapie sehr vorsichtig sein. / Therapists should be very careful regarding the use of modern technologies in psychotherapy.					
Es sollte eine strengere Regulierung der über moderne Technologien verfügbaren Inhalte in Bezug auf Psychotherapie und Psychopathologie durchgesetzt werden. / A stricter oversight of content available through Modern Technologies regarding psychotherapy and psychopathology should be enforced.					
Ein:e Therapeut:in sollte sich weigern, mit einem:einer Klient:in über moderne Technologien (WhatsApp, Instagram, Facebook etc.) in Verbindung zu treten. / A therapist should refuse to connect with a client via modern technologies (WhatsApp, Instagram, Facebook, etc.).					
In einigen Fällen können Moderne Technologien den therapeutischen Prozess beeinträchtigen. / In some cases,					

modern technologies can harm the therapeutic process.					
Ein:e erfahrene:r Therapeut:in hat keinen Bedarf an modernen Technologien. / An experienced therapist has no need for modern technologies.					
Moderne Technologien sind in der Psychotherapie unnötig. / Modern technologies are unnecessary in psychotherapy.					
Der Aufwand, sich über die Möglichkeiten des Einsatzes von modernen Technologien in der Psychotherapie zu informieren, würde sich nicht lohnen. / The effort to learn about possibilities of using Modern Technologies in psychotherapy would not pay off.					
Moderne Technologien bringen keine Vorteile, die über konventionelle therapeutische Techniken hinausgehen. / Modern technologies do not bring benefits beyond conventional therapeutic techniques.					
